# Needle size for thyroid fine needle aspiration cytology. A single institution experience

**DOI:** 10.3389/fsurg.2024.1368614

**Published:** 2024-07-05

**Authors:** Pasquale Cianci, Rocco Tumolo, Ivana Conversano, Damiano Travaglia, Giuseppe Trigiante, Giuliano Lantone, Vincenzo Lizzi, Miriam Cappiello, Marco Montagna, Fabio Pio Curci, Enrico Restini

**Affiliations:** ^1^ Department of Surgery and Traumatology, ASL BAT, Lorenzo Bonomo Hospital, Andria, Italy; ^2^ Department of Medical and Surgical Sciences, Azienda Ospedaliero-Universitaria Policlinico Riuniti, Foggia, Italy

**Keywords:** thyroid gland, fine needle aspiration, thyroid nodules, cytology, interventional ultrasonography

## Abstract

**Background:**

Fine needle aspiration cytology (FNAC) is an invasive diagnostic technique which is widely used for the cytological diagnosis of thyroid nodules. This procedure is generally widely tolerated by patients, albeit often accompanied by local pain and discomfort. Despite various proposals of execution methods, no approach is universally accepted,especially regarding the size of the needle to be used for sampling. Needle gauge preferences vary across regions, with 25-gauge needles more common in Western countries and 22-gauge needles favored in Asian countries. Complications associated with larger needles have been studied but remain inconclusive.

**Materials and methods:**

Over one year, we conducted 300 FNAC procedures under ultrasound guidance, employing both 22-gauge and 25-gauge needles. In no patient was local anesthesia performed before starting the procedure, which in all was performed by the same operator. Similarly the cytological examination of the material taken was performed by a single operator. Patients, 105 males and 195 females, were divided into two groups of 150 each based on the needle size used.

**Results:**

Patients treated with 22-gauge needles reported higher levels of pain during the procedure and increased discomfort afterward. Similarly, a greater incidence of hematomas and vasovagal reactions was noted in this group. However, the cell sample obtained and diagnostic response rates were consistent across both groups.

**Conclusion:**

On the basis of our observations we concluded that the size of the needle used is irrelevant for the purposes of the diagnostic result, as long as the procedure is performed by expert personnel. However, the 25-gauge needle is preferable because it's more tolerated and accepted by patients. Our results do not represent a single and conclusive verdict, but they could represent the starting point for further research.

## 1 Introduction

Thyroid fine needle aspiration cytology (FNAC) is a procedure first described in 1948 (1) that allows the collection of biological material through a very fine needle. This material is then subjected to cytological examination to determine whether it is benign or malignant in nature (2). This method represents the gold standard for the preoperative diagnosis of thyroid nodules because the cytological results obtained allow the identification of nodules with malignant features, which require surgical intervention, and those with benign features, which should be subjected to surveillance, thus reducing the number of thyroidectomies in patients with benign pathologies (3). Thyroid nodules are common, with a clinical prevalence estimated at around 3%–7% of the general population. They are more prevalent in females (4), and their incidence increases with age. The extensive use of ultrasound as a screening tool has demonstrated that many individuals harbor non-palpable nodules (5). In the presence of a thyroid nodule, clinical examination alone does not allow the distinction between benign and malignant nodules (6), and international guidelines suggest that further diagnostic procedures should be initiated in such patients. Bethesda System for Reporting Thyroid Cytopathology is currently the most used, in Italy in addition to this international classification there is another reporting system “The Italian thyroid cytology classification system” which was recently updated by the United Italian Society of Endocrine Surgery. The Italian classification system is more detailed regarding the percentage of malignancy risk (7, 8). In this context, ultrasound-guided FNAC is recognized as the most reliable diagnostic technique due to its high precision, safety, ease of execution, and cost-effectiveness (9). Over time, efforts have been made to improve both the methodology and the instrumentation used in FNAC. It is undeniable that the procedure, like all techniques, requires a learning curve for the operator, and its accuracy may differ among different operators based on different operational planning (5). Ideally, it is useful to seek ergonomics in the workplace, with the correct positioning of the patient and instruments, and finding the most suitable position for the assistant to achieve comfort in movements and avoid awkward crosses. To these subjective parameters, variables related to the equipment are added, such as the type of ultrasound column, the ultrasound probe, and the type and size of the needle. In particular, the needle size has been taken as a parameter for comparison to assess whether using needles of different gauges can improve the performance of the procedure. As for the needle size, in Western countries, needles of 25 or 27 G are primarily used, while in Eastern countries, the use of 21–22 G needles is common (3).

Few studies have compared the results obtained using needles of different gauges, and in most cases, the results are controversial, as other factors likely contribute to the determination of outcomes (10). Building on these premises, we wanted to initiate a study in our group where the results obtained using two different gauges of fine needles for the procedure were compared: needles with a gauge of 22 G and needles of 25 G.

## 2 Materials and methods

From June 2022 to June 2023, in our Department, we performed 300 ultrasound-guided FNAC for thyroid nodules. We used, alternatively for each patient, a 22-gauge or 25-gauge needle, until reaching a sample of 150 for each group. Ultrasound was performed using the TOSHIBA NEMIO SSA-550A unit with a linear probe PLM-703AT (Toshiba Medical Systems Corporation, Otawara-Shi, Tochigi-Ken, Japan). By choice, no local anesthetic was administered before the procedure, for aspiration we used a 5 ml syringe and we gave ourselves a limit of 3 attempts per patient. Following the indications of our pathologists, the aspirated materials were smeared and pressed onto the surface of 2 glass slides. After this application, the remaining part of the sample was processed by ThinPrep® 2000. Of the 300 patients observed, we treated 105 males (35%) and 195 females (65%) with a mean age of 54.01 years (range 16–81). The characteristics of the patients are shown in Table 1. We conducted our observations prospectively, assigning patients to one of the two groups with a 1 to 1 ratio. All procedures were performed by a single operator and a single cytopathologist, this to minimize biases due to the technique.

**Table 1:** Demographic data.

	Group 22G (*n* = 150)	Group 25G (*n* = 150)	*p*-value
Gender (*n*, %)			0.628
Male	55 (36.7)	50 (33.3)	
Female	95 (63.3)	100 (66.7)	
Age (year)			0.660
Mean (SD)	54.25 (13.85)	53.76 (13.64)	
Median	42.5	49	
Range	16–81	18–80	
BMI (kg/m^2^)			0.912
Mean (SD)	20 (4.28)	21.5 (4.54)	
Median	20	19	
Range	18–22	18–20	
Nodule size (mm)			0.764
Mean (SD)	25.29 (10.26)	23.92 (8.87)	
Median	22	22	
Range	11–55	10–56	

### 2.1 Statistical analysis

Statistical analysis was performed using SPSS (Statistical Package for Social Science, IBM SPSS Statistics, Version 28; IBM Corp., Armonk, NY, USA). Data are presented as number and percentage, with mean value and standard deviation, median and range. For bivariate two-sided comparisons between the Group 22G and the Group 25G, Chi-squared test or Fisher's exact test were used for categorical variables, whereas the Mann–Whitney U test was applied for continuous variables. Differences were considered significant at *p*-values <0.05.

## 3 Results

Of the 300 total patients, 150 were assigned to Group 22G and the same number to Group 25G. The assignment was conducted prospectively and with random alternation with a 1 to 1 ratio. As highlighted in Table 1, the demographic data (gender, age, BMI, nodule size) showed no differences in the 2 study groups. For complications related to the procedural technique, we have included both the most frequent variables and the less common events in Table 2. The differences found concerned the pain reported by the patients and the finding of hematoma. To detect pain, the VAS scale was used which for ease of calculation was divided into 2 levels (L1 0–5, L2 6–10). 98.7% of patients belonging to Group 25G reported L1 pain compared to 90% of Group 22G, on the other hand major pain (L2) was observed in 10% of cases of the latter group (*p*-value 0.002), denoting therefore a difference between the 2 samples in favor of Group 25G. The finding of hematoma (extrathyroidal, intrathyroidal) also highlighted differences in favor of the 25G Group (0.7%) compared to 4.6% of the other Group (*p*-value 0.037). Regarding the other minor complications, no differences were highlighted (Site infection, Vasovagal reaction, Vocal cord injury, Tracheal puncture, Dysphagia, Paresthesia). In Table 3 we have included the results obtained from the performed procedures. The mean value of the procedure time was the same (*p*-value 0.150), as well as the comparison of the samples obtained (*p*-value 0.906) and the cytology results divided into Bethesda categories (*p*-value 0.679) showed no differences. Our observations indicate that the only differences found were in patient-reported pain and the occurrence of postprocedural hematoma, and that the 25G needle offers fewer complications.

**Table 2:** Procedural complications.

	Group 22G (*n* = 150)	Group 25G (*n* = 150)	*p*-value
Pain (VAS scale) (*n*, %)			0.002
0–5	135 (90)	148 (98.7)	
6–10	15 (10)	2 (1.3)	
Hematoma (*n*, %)			0.037
Extrathyroidal	2 (1.3)	1 (0.7)	
Intrathyroidal	5 (3.3)	0 (0)	
Site infection (*n*, %)	1 (0.7)	1 (0.7)	1
Vasovagal reaction (*n*, %)	5 (3.3)	2 (1.3)	0.448
Vocal cord injury (*n*, %)	0	0	1
Tracheal puncture (*n*, %)	0	0	1
Dysphagia (*n*, %)	1 (0.7)	0	1
Paresthesia (*n*, %)	1 (0.7)	1 (0.7)	1

**Table 3:** Results of thyroid aspiration cytology using 22- and 25-gauge needles.

	Group 22G (*n* = 150)	Group 25G (*n* = 150)	*p*-value
Time of procedure (min.)			0.150
Mean (SD)	6.84 (1.73)	6.58 (1.56)	
Median	7	6	
Range	4–10	4–10	
Sample obtained (*n*, %)			0.906
1	90 (60)	88 (58.7)	
2–3	60 (40)	62 (41.3)	
Bethesda categories (*n*, %, Italian classification system)			0.679
I. Non diagnostic	10 (6.7) (Tir 1)	9 (6)	
II. Benign	115 (76.7) (Tir 2)	120 (80)	
III. Atypia of undetermined significance	15 (10) (Tir 3A)	16 (10.7)	
IV. Follicular neoplasm	0 (Tir 3B)	0	
V. Suspicious for malignancy	8 (5.3) (Tir 4)	5 (3.3)	
VI. Malignant	2 (1.3) (Tir 5)	0	

## 4 Discussion

FNAC is a simple procedure to perform, with a low cost-benefit ratio, characterized by high diagnostic accuracy, making it the gold standard for the preoperative diagnosis of thyroid nodules (3). However, it is essential to be aware that adverse events may occur during the procedure, and one should be prepared for the possible onset of complications. FNAC is generally well-accepted by patients, although in some particularly sensitive individuals, the sight of the ultrasound column, syringe with a needle, and ultrasound probe may induce anxiety, which can persist even after the procedure. Most patients report pain at the time of needle insertion, but no intervention is necessary for its intensity or duration (11). Quantifying the pain intensity using the VAS Scale, the subjective perception of discomfort varies widely, from a sensation of tenderness to actual pain, albeit transient. The severity of painful symptoms has been related to various factors: the needle diameter (12), the number of “punctures,” the time taken for the procedure, the location of the nodules (13), and, not least, the operator's experience and the patient's character (14). However, isolating a characteristic element is difficult, and there is no consensus in the literature, which is discordant and certainly inconclusive (10). In our experience, we observed differences in the two groups, as reported by others (12, 15). The group treated with a 22 G needle reported higher pain intensity during the procedure and a discomfort sensation that persisted beyond 24 h after the procedure. We did not find differences in the two groups regarding the time taken, and concerning the amount of biological material collected, the needle caliber used did not affect the adequacy of the sample for cytological examination (16–20). Microscopic evaluation was performed at the Pathological Anatomy Institute of the Hospital without any information regarding the caliber of the needle used for the sample aspiration, and we never received reports of insufficient material. Cytological evaluation was then performed based on the Bethesda System for Reporting Thyroid Cytopathology on these samples. No serious complications occurred during the procedure in both groups. We reported the occurrence of a hematomas (9) at the puncture site with a slight prevalence in the group treated with a 22 G needle. In this respect, the decision to interrupt the intake of anticoagulants or antiplatelet agents before performing FNAC is under discussion. Recent experiences show no statistical significance between patient groups in whom the procedure was performed without suspending the intake of anticoagulant or antiplatelet drugs and groups where drug intake was suspended. The final decision is left to the individual operator, who should decide based on the individual characteristics of patients due to the low risk of bleeding events after FNAC of thyroid nodules (21–24). Cases of rare but sometimes fatal thyroid bleeding after FNAC requiring intubation or surgical intervention have been reported. Bonsignore et al. (25) reported the case of a 78-year-old woman who died due to a hematoma causing tracheal compression, and they conducted a literature review of 12 cases of severe thyroid bleeding, mostly managed with surgical interventions or conservative measures (25, 26). Vasovagal reactions are rare (27, 28). In our experience, they were more frequent in the 22G group, but mild and transient. Only one patient had a severe syncopal episode, requiring Trendelenburg positioning and prompt saline infusion. No structures adjacent to the puncture site were affected in either group. Only one patient in the 22G group reported dysphagia, while paresthesia at the puncture site and a mild infection at the puncture site were reported by four patients, two in each group. No patient experienced vocal cord injury or tracheal puncture. Dysphonia has been reported in some studies (22, 29). Possible mechanisms of recurrent laryngeal nerve palsy after FNAC have been attributed to rapid stretching of the nerve caused by a thyroid hematoma or direct nerve injury with the needle (30). This study certainly has a limitation, we only used 2 gauges for the needles during the procedures, leaving out those of 21, 23 and 27 gauge (15, 31, 32). The high costs and the difficulty in finding them influenced our choices, however in the near future it is our intention use them to have a broader and more conclusive comparison.

## 5 Conclusion

Based on our experience, as well as literature reports (12), it can be stated that FNAC is a safe technique with a low and not particularly relevant complication rate, not discouraging the execution of the procedure in the preoperative diagnosis of thyroid nodules. Especially concerning individual tolerance, we observed that the procedure performed with 25 G caliber needles is more accepted by patients, especially regarding subjective pain sensitivity. Like all procedures, however, it must be performed with caution, adopting all precautions to minimize adverse events that, although minimal, are always bothersome for patients (7). Our results represent only a single experience and certainly cannot be considered conclusive, but they could represent the starting point for further research.

## Data Availability

The raw data supporting the conclusions of this article will be made available by the authors, without undue reservation.
